# Hemoglobin to red cell distribution width ratio as a prognostic marker for ischemic stroke after mechanical thrombectomy

**DOI:** 10.3389/fnagi.2023.1259668

**Published:** 2023-11-21

**Authors:** Xianrong Feng, Yaodan Zhang, Qizheng Li, Baojia Wang, Jie Shen

**Affiliations:** ^1^Department of Neurology, Hospital of Chengdu University of Traditional Chinese Medicine, Chengdu, Sichuan, China; ^2^Department of Neurology, Chengdu Second People’s Hospital, Chengdu, Sichuan, China

**Keywords:** acute ischemic stroke, mechanical thrombectomy, hemoglobin to red cell distribution width ratio, poor prognosis, death, marker

## Abstract

**Background:**

The hemoglobin to red cell distribution width ratio (HRR) has been experimentally associated with the prognosis of acute ischemic stroke (AIS). However, its relationship with mechanical thrombectomy (MT) for AIS remains unclear. Therefore, this study aimed to investigate the relationship between HRR at admission, follow-up HRR, and clinical outcomes in patients undergoing MT.

**Methods:**

Acute ischemic stroke patients undergoing MT were consecutively enrolled from January 2017 to December 2022. Demographic, clinical, and laboratory information were collected. HRR was measured by dividing hemoglobin (Hb) by red cell distribution width (RDW) at admission and after 24 h of MT. Clinical outcomes after 3 months were evaluated using the modified Rankin Scale (mRS). The primary outcome was poor prognosis (mRS > 2) at 3 months, while the secondary outcome was death within 3 months.

**Results:**

A total of 310 patients were analyzed, of whom 216 patients (69.7%) had poor prognosis, and 92 patients (29.6%) died. Patients with a poor prognosis and death had significantly lower HRR levels at admission and after 24 h. HRR at admission was not associated with clinical outcomes according to multivariable logistic regression analysis. However, HRR after 24 h was significantly associated with poor prognosis (adjusted odds ratio [OR]: 0.646, 95% confidence interval [CI]: 0.520–0.803, *p* < 0.001) and death (adjusted OR: 0.615, 95% CI: 0.508–0.744, *p* < 0.001). Receiver-operating characteristic curve analysis demonstrated the predictive ability of HRR after 24 h, with areas under the curves of 0.790 for poor prognosis and 0.771 for death.

**Conclusion:**

Rapidly measurable HRR levels are an independent marker of outcome after MT in AIS patients. This may provide a reliable auxiliary outcome measure for clinical routine and interventional therapy.

## 1 Introduction

The Global Burden of Disease Report identifies stroke as the second leading cause of worldwide mortality and disability (Krishnamurthi et al., 2020). Mechanical thrombectomy (MT) has emerged as one of the most efficacious therapies for acute ischemic stroke (AIS; Powers et al., 2018, 2019). Timely and effective thrombectomy significantly improves the prognosis for AIS patients. However, complications such as cerebral hemorrhage, vascular re-occlusion, and cerebral edema may occur after MT, and the limited treatment time window presents challenges to treatment efficacy (Kimberly et al., 2018; Luby et al., 2023; Sarraj et al., 2023). Hence, it is crucial to investigate simple, convenient, and effective clinical indicators that can predict the prognosis of AIS, guide clinical decision-making, and enhance treatment outcomes.

Hemoglobin (Hb) and red cell distribution width (RDW) are conventional blood test parameters. Hb levels determine the oxygen-carrying capacity, while RDW quantifies the variation in red blood cell size (Lippi and Plebani, 2014; Salvagno et al., 2015). These parameters not only reflect the balance between hematopoietic function and red blood cell survival but also play a critical role in inflammation, oxidative stress, and the vascular innate immune system (Emans et al., 2011; Patel et al., 2013; Kawabata, 2020). For instance, anemia has been shown to induce the release of interleukin-6 and tumor necrosis factor-α (Feret et al., 2022), and RDW has been linked to inflammatory markers and oxidative stress (Förhécz et al., 2009). Inflammation and oxidative stress can worsen cerebral edema and hemorrhage, delay cerebral ischemia, and contribute to poor prognosis (Zhao et al., 2017; Esposito et al., 2022; Westendorp et al., 2022). These findings suggest that both Hb and RDW, as essential markers of underlying inflammatory processes, may be correlated with the clinical outcome of ischemic stroke. Hb levels have been identified as significant predictors of AIS and coronary heart disease (Kwok et al., 2016; Chang et al., 2020; Zhang et al., 2021). Research has also demonstrated that RDW can predict adverse outcomes in patients with acute myocardial infarction receiving percutaneous coronary intervention (PCI) and in patients with AIS (Feng et al., 2017; Parizadeh et al., 2019; Xiao et al., 2022). Furthermore, RDW, regardless of anemia status, has been associated with stroke severity and adverse outcomes in AIS, thereby improving stroke prediction accuracy (Saliba et al., 2015; Xue et al., 2022).

Recently, the hemoglobin-to-red cell distribution width ratio (HRR) has emerged as a novel biomarker for cardiovascular diseases. It is calculated from Hb and RDW without additional costs (Rahamim et al., 2022; Xiu et al., 2022). HRR has been studied in relation to myocardial infarction stenting and ischemic stroke associated with atrial fibrillation (Qin et al., 2022; Sun et al., 2022). However, further investigation is needed to determine whether HRR can effectively predict the prognosis of AIS patients undergoing thrombectomy. Additionally, various components of the immune system undergo dynamic changes after AIS, which may have varying effects depending on the stage of stroke development. Therefore, our study aims to investigate the association between HRR and the prognosis of AIS patients undergoing thrombectomy, as well as explore the optimal time point at which HRR functions as a prognostic marker.

## 2 Materials and methods

### 2.1 Study population

From January 2017 to December 2022, this retrospective study included consecutive patients who underwent MT at Chengdu Second People’s Hospital, based on a prospective database. The MT selection criteria and time window strictly adhered to the current guidelines of the American Heart Association/American Stroke Association for the Early Management of Acute Ischemic Stroke Patients (Powers et al., 2015, 2018; Greenberg et al., 2022). The choice of materials and thrombectomy approach, whether stent-retriever, aspiration, or a combined technique, was determined by the neuro-interventionalist. Follow-up computed tomography (CT) scans were performed approximately 24 h after MT to assess any intracranial hemorrhage.

**Figure 1:**
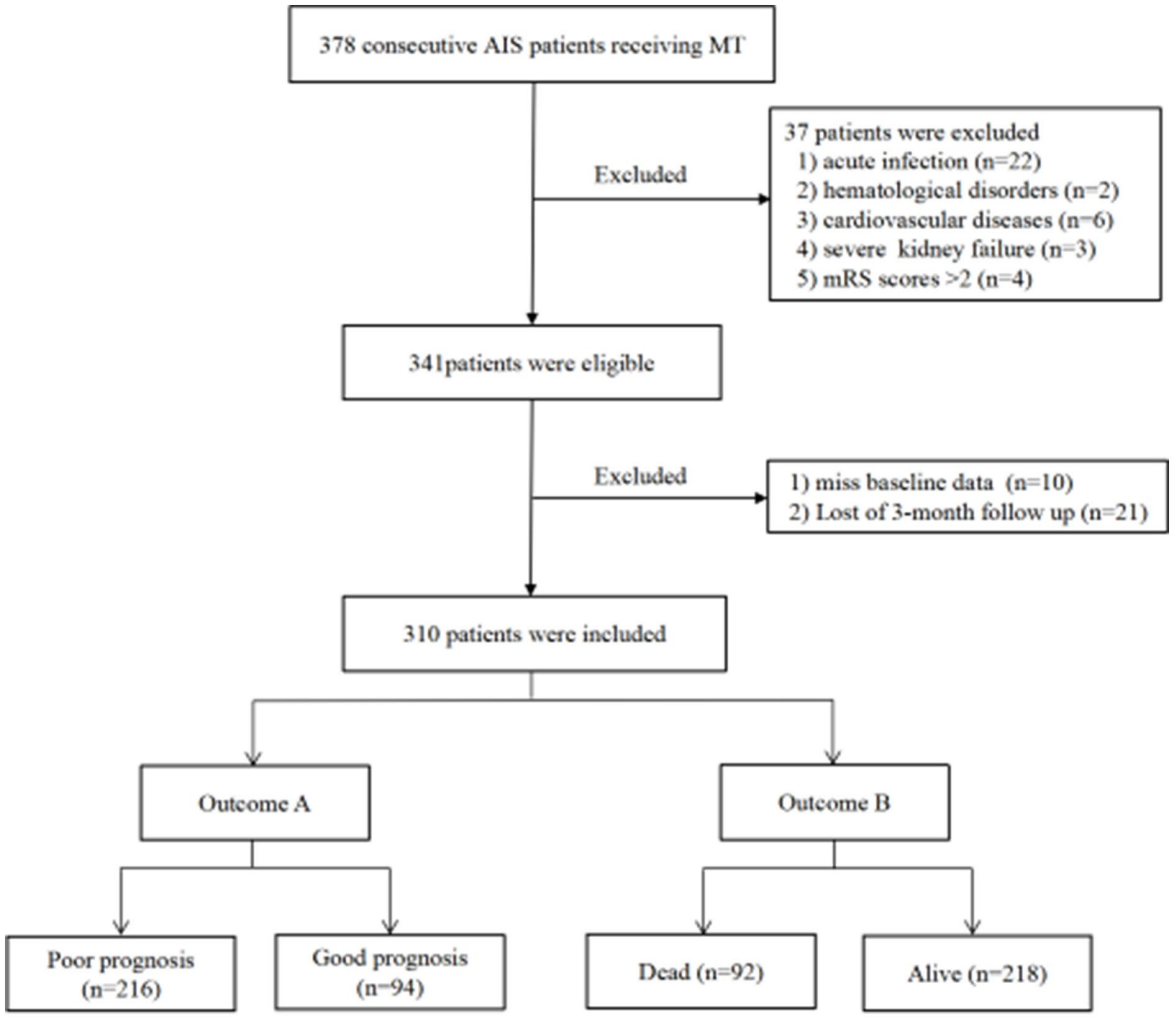
Study flow chart. AIS, Acute ischemic stroke; MT, Mechanical thrombectomy; mRS, Modified Rankin scale.

The exclusion criteria for this study were as follows: (1) acute or chronic infection; (2) severe systemic illnesses such as malignancy, hematological disorders, severe heart failure, liver, or renal dysfunction; (3) incomplete clinical data; (4) modified Rankin Scale (mRS) score > 2 prior to the onset of stroke; and (5) patients who were lost to follow-up. 378 AIS patients who underwent MT were initially screened, and 37 patients were excluded based on the inclusion and exclusion criteria. Additionally, 21 patients were lost during the follow-up period. After excluding 10 patients who lacked baseline Hb and RDW values, the final sample size for the analysis was 310 patients (Figure 1). The retrospective study obtained approval from the ethics committee of Chengdu Second People’s Hospital, and patients or their families provided written informed consent.

### 2.2 Data collection

Demographic information, past medical history, vascular risk factors, National Institutes of Health Stroke Scale (NIHSS) scores at admission, pre-admission modified Rankin Scale (mRS) scores, results of computed tomography angiography or digital subtraction angiography, pre-treatment magnetic resonance imaging, and information regarding whether intravenous thrombolysis was administered before MT and post-MT recanalization rates were obtained from medical institution databases for analysis. Stroke etiology was classified according to the Trial of Org 10172 in Acute Stroke Treatment (TOAST) criteria (Adams et al., 1993). Blood samples were collected at admission and 24 h after MT to measure Hb and RDW levels. The HRR was calculated as the ratio of Hb to RDW. CT scans were conducted 24 h after MT to evaluate symptomatic intracranial hemorrhage (sICH). Follow-up data on current mRS scores were obtained through standardized telephone interviews from symptom onset to 3 months later.

### 2.3 Clinical outcomes

Successful reperfusion was defined as the modified Thrombolysis in Cerebral Infarction (mTICI) ≥ 2b (Mokin et al., 2014; Kaesmacher et al., 2018). sICH was defined as intracranial hemorrhage with an increase of at least four points on the NIHSS scale (Hacke et al., 1998). The modified Rankin Scale (mRS) was used to assess the primary outcome at 3 months; good prognosis was defined as mRS ≤ 2, while poor prognosis was defined as mRS > 2 (Hill et al., 2020; Rebchuk et al., 2020). The secondary outcome was death within 3 months of MT. Death was defined as an all-cause passing resulting from a stroke.

### 2.4 Statistical analysis

All data were analyzed using SPSS version 22.0 (SPSS Inc., Chicago, IL, United States), GraphPad Prism version 8.0 (GraphPad Software, San Diego, California, United States), and MedCalc version 22.0 (MedCalc Software, Ostend, Belgium). The Kolmogorov–Smirnov test assessed the distributional normality. Categorical variables were presented as counts and percentages, and compared using the chi-squared test, while continuous variables were presented as mean ± standard deviation or median with interquartile range (IQR). Multivariate logistic regression was used to examine the impact of HRR on outcomes, adjusted for variables selected through the forward selection method, calculating odds ratios (ORs), and 95% confidence intervals (CIs). The Pearson correlation test was utilized to analyze the correlation between baseline laboratory data and data collected after 24 h. The discrimination of Hb, RDW, and HRR for outcomes was analyzed using the area under the receiver operating characteristic curve (AUC-ROC). Cut-off values for each biomarker were determined by Youden index. The generalized linear model was obtained by binary logistic regression analysis combining different parameters. Prediction probability was calculated from the regression equation as an additional parameter, which was further evaluated by ROC analysis. *p* < 0.05 was regarded as statistically significant.

## 3 Results

### 3.1 Basic characteristics of study patients

The study included a total of 310 patients who underwent MT (Figure 1). The detailed characteristics of the patients are shown in Table 1. The median age of the patients was 72 (60–79) years, with 139 (44.8%) being female. The median NIHSS score at baseline was 15 (12–20). Prior to MT, 86 patients (27.7%) received intravenous thrombolysis. Successful reperfusion (mTICI ≥ 2b) was achieved in 82.3% of cases following MT. After MT, sICH was observed in 39 patients (12.6%). At 3 months, 216 patients (69.7%) had a poor prognosis, and 92 patients (29.6%) died. In addition, significant statistical differences were found between patients with good and poor prognosis concerning age, sex, atrial fibrillation, baseline NIHSS score at admission, NIHSS score after 24 h, sICH, and successful reperfusion (*p* < 0.05). Those who died within 3 months were more likely to be female, had older age, a higher NIHSS score at admission and after 24 h, a higher incidence of sICH, and a lower success rate of reperfusion (*p* < 0.05).

**Table 1:** Basic characteristics of study population according to 3-month prognosis and occurrence of death.

Variables	Total (*N* = 310)	Good prognosis (*N* = 94)	Poor prognosis (*N* = 216)	*p*	Alive (*N* = 218)	Dead (*N* = 92)	*p*
Demographic data							
Age, years, median (IQR)	72 (60–79)	64 (54–75)	74 (63–81)	<0.001	70 (58–78)	76 (66–81)	0.003
Sex (female), *n* (%)	139 (44.8)	29 (30.9)	110 (50.9)	0.001	88 (40.4)	51 (55.4)	0.018
Stroke risk factors, *n* (%)							
Hypertension	166 (53.5)	49 (52.1)	117 (54.2)	0.805	123 (56.4)	43 (46.7)	0.135
Diabetes mellitus	55 (17.7)	14 (14.9)	41 (19.0)	0.423	35 (16.1)	20 (21.7)	0.256
History of stroke	39 (12.6)	11 (11.7)	28 (13.0)	0.853	27 (12.4)	12 (13.0)	0.853
Hyperlipidemia	38 (12.3)	12 (12.8)	26 (12.0)	0.852	25 (11.5)	13 (14.1)	0.570
Atrial fibrillation	160 (51.6)	33 (35.1)	127 (58.8)	<0.001	109 (50.0)	51 (55.4)	0.387
Smoking	95 (30.6)	35 (37.2)	60 (27.8)	0.108	70 (32.1)	25 (27.2)	0.421
Drinking	59 (19.0)	23 (24.5)	36 (16.7)	0.117	46 (21.1)	13 (14.1)	0.205
Antiplatelet agents	30 (9.7)	11 (11.7)	19 (8.8)	0.412	23 (10.6)	7 (7.6)	0.530
Anticoagulant	27 (8.7)	5 (5.3)	22 (10.2)	0.193	18 (8.3)	9 (9.8)	0.663
Stroke evaluation							
Baseline NIHSS score, median (IQR)	15 (12–20)	13 (9–16)	17 (13–20)	<0.001	14 (11–18)	17 (14–22)	<0.001
Intravenous thrombolysis, *n* (%)	86 (27.7)	23 (24.5)	63 (29.2)	0.412	60 (27.5)	26 (28.3)	0.890
Location of stroke, *n* (%)				0.196			0.712
Anterior circulation	270 (87.1)	78 (83.0)	192 (88.9)		191 (87.6)	79 (85.9)	
Posterior circulation	40 (12.9)	16 (17.0)	24 (11.1)		27 (12.4)	13 (14.1)	
Symptom onset to treatment time, min	254 (187–304)	251 (174–301)	256 (189–306)	0.636	243 (182–302)	261 (194–316)	0.091
mTICI ≥2b, *n* (%)	255 (82.3)	90 (95.7)	165 (76.4)	<0.001	191 (87.6)	64 (69.6)	<0.001
Pre-mRS	0 (0–0)	0 (0–0)	0 (0–0)	0.648	0 (0–0)	0 (0–0)	0.935
TOAST, *n* (%)				0.402			0.491
Large-artery atherosclerosis	173 (55.8)	47 (50.0)	126 (58.3)		122 (56.0)	51 (55.4)	
Small-vessel occlusion	109 (35.2)	39 (41.5)	70 (32.4)		78 (35.8)	31 (33.7)	
Cardioembolism	10 (3.2)	2 (2.1)	8 (3.7)		8 (3.7)	2 (2.2)	
Other determined/undetermined	18 (5.8)	6 (6.4)	12 (5.6)		10 (4.6)	8 (8.7)	
NIHSS score after 24 h, median (IQR)	15 (8–21)	6 (3–11)	18 (12–25)	<0.001	12 (6–18)	23 (16–36)	<0.001
Outcomes, *n* (%)							
90-day mRS	4 (2–6)	1 (0–1)	5 (4–6)	<0.001	3 (1–4)	6 (6–6)	<0.001
sICH	39 (12.6)	1 (1.1)	38 (17.6)	<0.001	17 (7.8)	22 (23.9)	<0.001

Continuous and categorical variables were compared using the Mann–Whitney U-test and chi-squared test. NIHSS, National Institutes of Health Stroke Scale; mRS, Modified Rankin Scale; mTICI, Modified Thrombolysis in Cerebral Infarction; sICH, Symptomatic intracranial hemorrhage; and IQR, Interquartile range.

### 3.2 Association of lower HRR with poor prognosis

**Figure 2:**
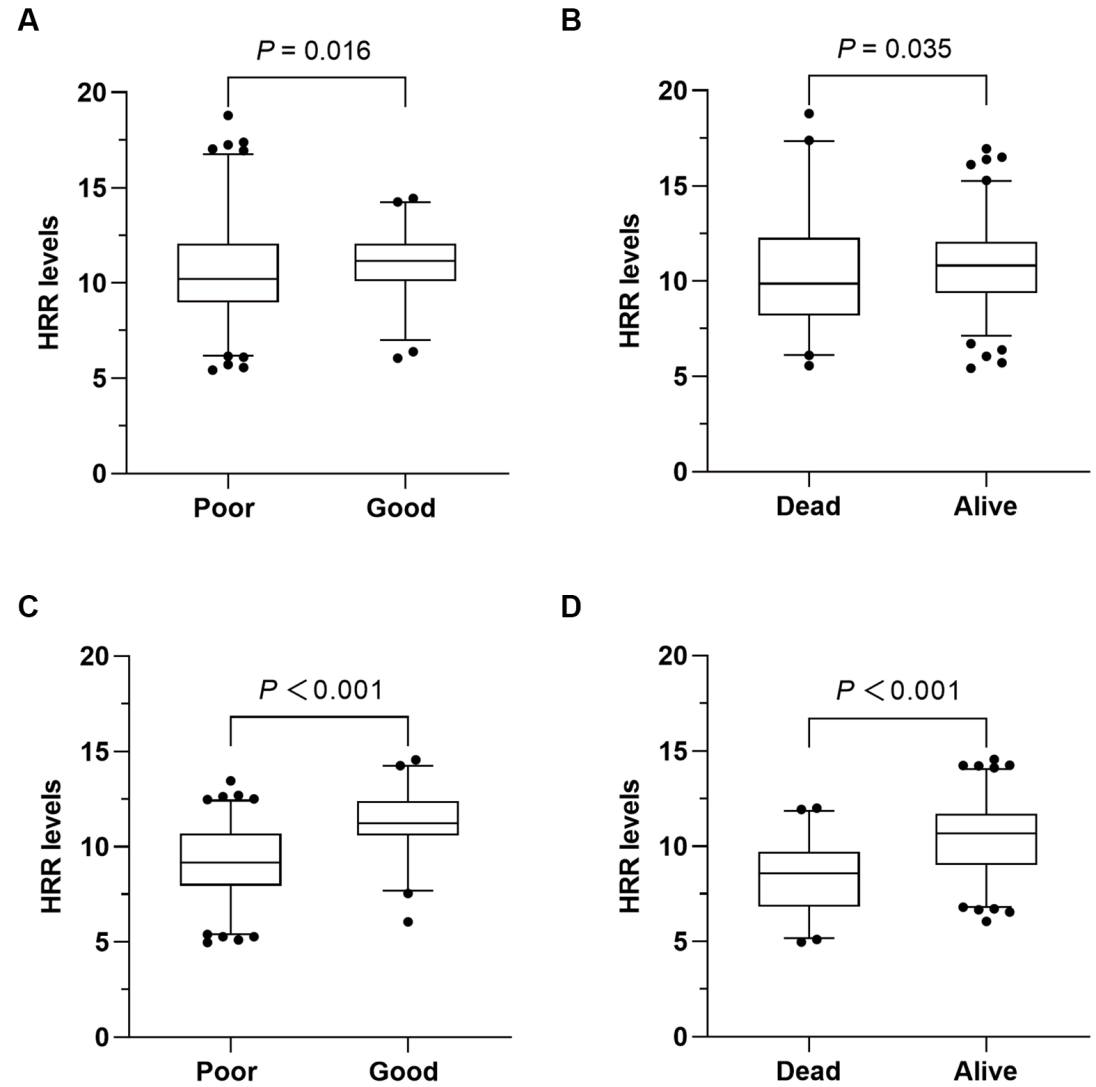
Comparison of HRR levels between different outcomes. **(A,B)** Comparison of hemoglobin to red cell distribution width ratio (HRR) at admission according to different outcomes. **(C,D)** Comparison of HRR after 24 h according to different outcomes.

At admission, no differences in Hb and RDW levels were observed between patients with good prognosis and those without. However, patients with poor prognosis had lower HRR levels compared to those with a good prognosis [10.22 (8.97–12.05) vs. 11.18 (10.09–12.06); *p* = 0.016; Table 2; Figure 2A]. Nevertheless, multivariate analysis indicated that HRR at admission showed no correlation with the prognosis after 3 months in AIS patients undergoing MT (OR = 1.045; 95% CI: 0.886–1.232; *p* = 0.605; Table 3).

**Table 2:** Comparison of HRR laboratory data according to different outcomes.

Variables	Good prognosis	Poor prognosis	*p*	Alive	Dead	*p*
At admission						
Hb	121 (97–141)	119 (96–145)	0.810	121 (97–145)	118 (93–143)	0.279
RDW	11.1 (9.0–13.3)	11.4 (9.6–13.5)	0.249	11.2 (9.3–13.6)	11.3 (9.8–13.0)	0.667
HRR	11.18 (10.09–12.06)	10.22 (8.97–12.05)	0.016	10.82 (9.37–12.04)	9.87 (8.21–12.27)	0.035
After 24 h						
Hb	121 (97–140)	119 (93–143)	0.460	120 (95–143)	118 (88–139)	0.120
RDW	10.8 (9.0–12.9)	13.0 (10.9–14.9)	<0.001	11.8 (9.4–13.9)	13.6 (11.9–15.8)	<0.001
HRR	11.24 (10.60–12.40)	9.18 (7.95–10.70)	<0.001	10.67 (9.01–11.71)	8.57 (6.83–9.70)	<0.001

Variables were compared using the Mann–Whitney U-test. Values are presented as median (IQR). Hb, Hemoglobin; RDW, Red cell distribution width; HRR, Hemoglobin to red cell distribution width ratio.

**Table 3:** Multivariable analysis of HRR data in predicting clinical outcomes.

Variable	β	SE	Adjusted OR	(95% CI)	*p*
Poor prognosis^a^					
HRR at admission	0.044	0.084	1.045	0.886–1.232	0.605
HRR after 24 h	−0.437	0.111	0.646	0.520–0.803	<0.001
Occurrence of death^b^					
HRR at admission	0.022	0.062	1.022	0.905–1.154	0.728
HRR after 24 h	−0.487	0.097	0.615	0.508–0.744	<0.001

Multivariate analysis was performed using logistic regression analysis. ^a^HRR after 24 h: adjusted age, NIHSS score after 24 h, mTICI ≥ 2b. HRR at admission: sICH was additionally adjusted, along with above factors. ^b^HRR after 24 h: adjusted age, hypertension, NIHSS score after 24 h, and antiplatelet agents. HRR at admission: adjusted age, hypertension, NIHSS score after 24 h, and mTICI ≥ 2b.

After 24 h of MT, a significant difference in RDW was observed [13 (10.9–14.9) vs. 10.8 (9.0–12.9); *p* < 0.001; Table 2], while there was no difference in Hb levels between patients with good and poor prognosis. And HRR levels were significantly lower in patients with poor prognosis [9.18 (7.95–10.70) vs. 11.24 (10.60–12.40); *p* < 0.001; Table 2; Figure 2C]. In multivariate logistic regression, lower HRR was independently associated with a higher risk of poor prognosis (OR = 0.646; 95% CI: 0.520–0.803; *p* < 0.001; Table 3). Furthermore, the multivariate analysis revealed that older age, higher NIHSS score after 24 h, and lower successful reperfusion rate were risk factors for poor prognosis (Supplementary Table 1).

In addition, we observed strong and statistically significant positive correlations between Hb at admission and after 24 h (*r* = 0.983, *p* < 0.001), between RDW at admission and after 24 h (*r* = 0.703, *p* < 0.001), as well as a moderate and positive correlation between HRR at admission and after 24 h (*r* = 0.507, *p* < 0.001).

### 3.3 Association of lower HRR with death

Similarly, the HRR levels exhibited a significant difference between dead and alive patients at admission [9.87 (8.21–12.27) vs. 10.82 (9.37–12.04); *p* = 0.035; Table 2; Figure 2B], as well as after 24 h [8.57 (6.83–9.70) vs. 10.67 (9.01–11.71); *p* < 0.001; Table 2; Figure 2D]. After adjusting for potential confounding variables, HRR after 24 h was found to be an independent predictor of death (OR 0.615; 95% CI 0.508–0.744; *p* < 0.001; Table 3). However, HRR at admission was not associated with death (OR = 1.022; 95% CI: 0.905–1.154; *p* = 0.728; Table 3). Other risk factors for death were showed in Supplementary Table 2.

### 3.4 ROC curve analysis of HRR as a prognostic marker

**Figure 3:**
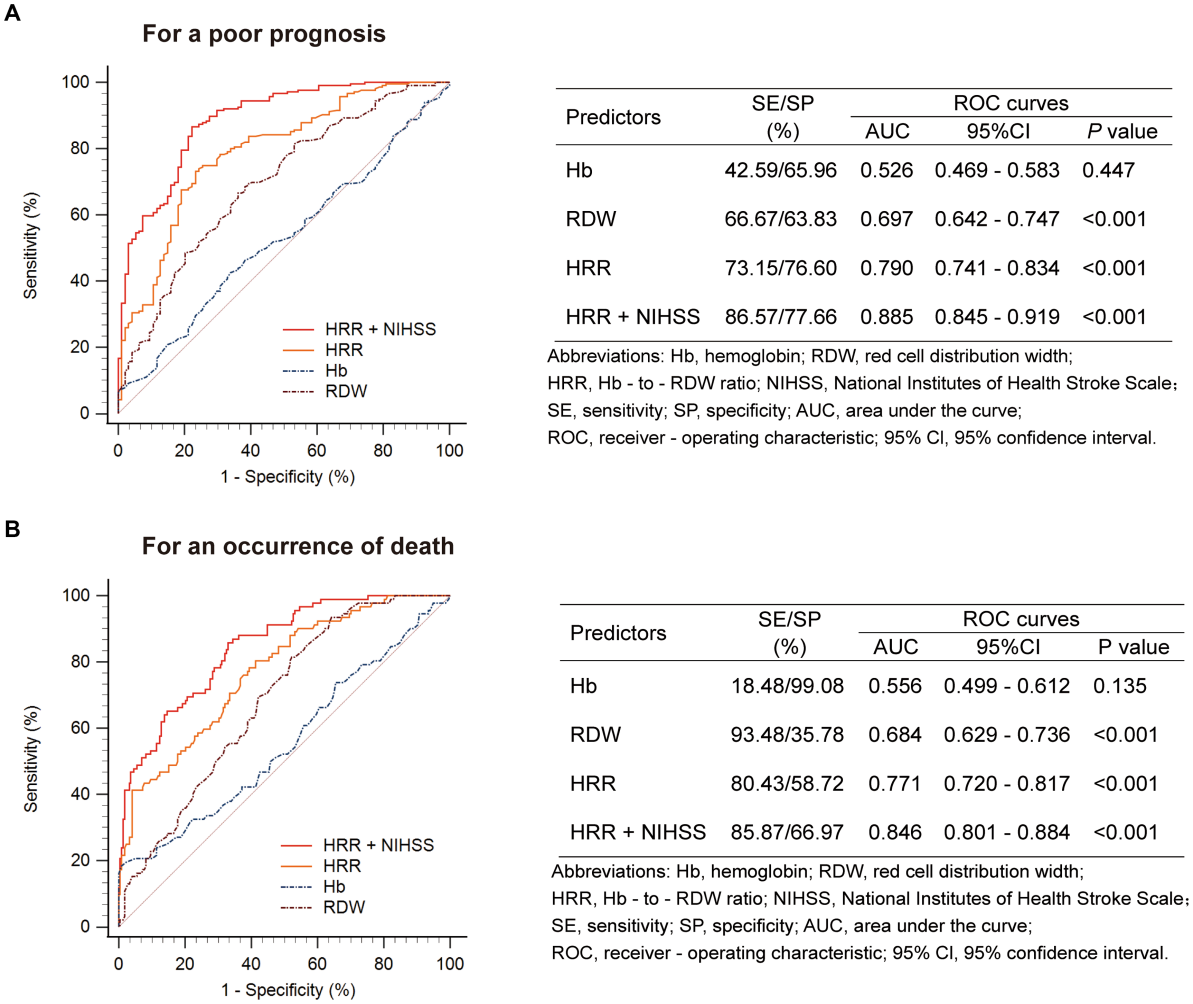
Prognostic accuracies of HRR as a predictor of **(A)** a poor prognosis or **(B)** an occurrence of death at 3 months after stroke.

Receiver operating characteristic analysis showed that HRR after 24 h was more discriminative of clinical outcome (poor prognosis and death) than HRR at admission (Supplementary Figure 1). Given that the results showed significant differences in HRR at 24 h among patients with poor prognosis, as well as the association between Hb, RDW, and HRR, we used ROC curves to evaluate their roles as prognostic markers (Figure 3A). Among all patients, RDW had good diagnostic accuracy in predicting poor prognosis, with an AUC of 0.697 (95% CI: 0.642–0.747, *p* < 0.001), sensitivity of 66.67%, and specificity of 63.83%. Notably, as a composite predictor calculated from Hb and RDW, HRR demonstrated even stronger discriminative ability, with an AUC of 0.790 (95% CI: 0.741–0.834, *p* < 0.001), sensitivity of 73.15%, and specificity of 76.60%. Furthermore, when combined with HRR, the AUC value of current NIHSS score for predicting poor prognosis increased from 0.859 (95% CI: 0.815–0.896) to 0.885 (95% CI: 0.845–0.919) (*p* = 0.025), with a sensitivity of 86.57% and specificity of 77.66%.

Similar results were found when evaluating the predictive value of HRR for death (Figure 3B).

## 4 Discussion

The purpose of this study was to investigate the association between the novel biomarker HRR and the outcomes of AIS patients who underwent MT. Our findings suggested that HRR at admission and after 24 h may be significantly lower in patients with poor prognosis and death. However, after adjusting for cerebrovascular risk factors, HRR after 24 h remained an independent risk factor for both poor prognosis and death, regardless of whether intravenous thrombolysis was performed prior to MT.

Anemia is a prevalent condition among AIS patients, and its pathogenesis involves various mechanisms such as reduced oxygen-carrying capacity, inflammation, energy imbalance, and hypercoagulation (Chang et al., 2020). Decreased levels of Hb imply a compromised oxygen-carrying capacity, leading to limited oxygen supply and an energy imbalance in the ischemic penumbra (Kimberly et al., 2013; Desai et al., 2023). Moreover, anemia can trigger the release of inflammatory factors like tumor necrosis factor-α (Feret et al., 2022). Recently, the role of inflammatory reactions in the progression of AIS and its association with poor prognosis has been widely recognized (Kehrel and Fender, 2016; Zhao et al., 2017; Shi et al., 2019; Esposito et al., 2022). Studies have indicated that overall poor nutritional status and weakened immune response may contribute to a worse prognosis (Bullock et al., 2020). Furthermore, anemic patients may experience hypercoagulability, further increasing the risk of ischemic events. Evidence suggests that severe anemia substantially raises the likelihood of stent thrombosis (Pilgrim et al., 2012), and lower Hb level was a strong predictor of post-PCI in-stent restenosis (Hu et al., 2021). Several studies have highlighted baseline Hb as a significant predictor of mortality and ischemic events in PCI (Reinecke, 2003; Dutsch et al., 2022). Additionally, a U-shaped association has been found between Hb levels and poor prognosis, all-cause mortality, and stroke severity in ischemic stroke (Chang et al., 2020; Zhang et al., 2021). Another study reported that lower Hb levels and variability were linked to mortality at 3 months in AIS patients undergoing MT (Nisar et al., 2021). In our investigation, AIS patients undergoing MT with poor prognosis and death had lower Hb levels compared to those with good prognosis and survival, although the difference was not statistically significant.

The value of RDW in cardiovascular disease reflects various mechanisms in the pathophysiological process. Firstly, an increased RDW indicates an underlying inflammatory state and impaired maturation of red blood cells (Ridker et al., 2001). RDW has been associated with inflammatory markers such as C-reactive protein with high sensitivity, interleukin-6, and fibrinogen levels (Förhécz et al., 2009). These inflammatory factors can disrupt red blood cell maturation by affecting the homeostasis of red blood cells, impairing iron metabolism, and inhibiting erythropoietin production (Rechavi and Rivella, 2008; Salvagno et al., 2015). Secondly, oxidative stress and microcirculatory damage play a significant role. Red blood cells possess substantial antioxidant capacity, and oxidative damage can lead to decreased cell survival rates (Emans et al., 2011; Lippi and Plebani, 2014). Changes in the morphology of red blood cells are associated with oxidative stress (Friedman et al., 2004). In addition, reduced red blood cell deformability can impede blood flow through the microcirculation, exacerbating ischemic conditions (Patel et al., 2013). Several studies have independently linked higher baseline RDW levels to mortality and adverse cardiovascular events in myocardial infarction patients undergoing PCI (Fatemi et al., 2013; Bujak et al., 2015; Zhao et al., 2015; Xiao et al., 2022). Research has also demonstrated that RDW may serve as a significant prognostic factor in AIS (Feng et al., 2017; Mohindra et al., 2020). Baseline RDW has been proposed as a prospective marker of mortality in AIS patients undergoing intravenous thrombolysis (Pinho et al., 2018; Ye et al., 2020). Similarly, in our study, AIS patients with poor prognosis and mortality tended to have elevated RDW levels 24 h after MT.

Although Hb and RDW have shown prognostic value in AIS patients, these two parameters can be influenced by various factors as mentioned above. Since HRR is calculated from Hb and RDW, it may provide a more effective and stable assessment compared to individual Hb or RDW measurements, objectively reflecting inflammatory and microcirculatory status, thus potentially serving as a superior biomarker. HRR has recently emerged as a crucial indicator for predicting cardiovascular disease mortality and prognosis (Rahamim et al., 2022). Lower HRR is associated with a higher risk of frailty and adverse outcomes in hospitalized older patients with coronary heart disease (Qu et al., 2021). Moreover, HRR may serve as a reliable indicator of mortality risk in patients with coronary artery disease after PCI (Sun et al., 2022; Xiu et al., 2022). Lower HRR levels have been found to increase the risk of death from all causes in AIS patients with atrial fibrillation (Qin et al., 2022). However, no studies have investigated the predictive significance of HRR for clinical outcomes in AIS patients undergoing MT. By excluding the influence of cardiovascular diseases, malignancies, infectious diseases, serious liver and kidney dysfunction, and adjusting for multivariate confounders, our study suggests that lower HRR significantly increases the risk of poor prognosis and death in AIS patients undergoing MT. Additionally, HRR demonstrates superior discriminative accuracy compared to the single parameter RDW. Globally, the NIHSS score is recognized as a valuable prognostic indicator for AIS, and incorporating HRR into the NIHSS enhances its predictive value.

While our study investigated the association between HRR and outcomes in AIS patients undergoing MT, we acknowledge certain limitations. Firstly, we only collected HRR data at admission and after 24 h of MT, precluding analysis of HRR changes throughout the entire stroke process. Secondly, there was a loss to follow-up of 21 patients, potentially introducing bias into our results. Thirdly, this was a retrospective and single-center design study, raising the possibility of selection bias and limiting the generalizability of our findings. It is important to consider these limitations when interpreting the results, and future research should address these issues to provide more robust evidence on the relationship between HRR and outcomes in AIS patients undergoing MT.

## 5 Conclusion

Our study indicates that HRR levels after 24 h of MT, as a simple, novel, cost-effective, and valuable biomarker, are an independent predictor of poor prognosis and death for AIS patients undergoing MT. However, further research is necessary to elucidate the underlying biological mechanisms and confirm the clinical utility of HRR.

## Data availability statement

The raw data supporting the conclusions of this article will be made available by the authors, without undue reservation.

## Ethics statement

The studies involving humans were approved by the ethics committee of Chengdu Second People’s Hospital. The studies were conducted in accordance with the local legislation and institutional requirements. The participants provided their written informed consent to participate in this study.

## Author contributions

XF: Methodology, Data curation, Investigation, Writing – original draft, Funding acquisition. YZ: Investigation, Formal Analysis, Writing – review & editing. QL: Formal Analysis, Writing – review & editing, Software, Validation. BW: Writing – review & editing, Validation, Formal Analysis, Software. JS: Validation, Writing – review & editing, Project administration, Conceptualization, Methodology, Resources.
