# Supplementary material for: Hemoglobin to red cell distribution width ratio as a prognostic marker for ischemic stroke after mechanical thrombectomy
Source: Front Aging Neurosci. 2023 Nov 21;15:1259668. doi: 10.3389/fnagi.2023.1259668 (PMC10710154; doi:10.3389/fnagi.2023.1259668)
Supplement: Supplementary file 1 [file Data_Sheet_1.PDF]

# Supplementary Material

## 1 Supplementary Figures and Tables

### 1.1 Supplementary Table1. Multivariable analysis results for predicting poor prognosis.

| Variable | HRR after 24 hours | | | | | HRR at admission | | | | |
|---|---|---|---|---|---|---|---|---|---|---|
| | β | SE | Adjusted OR | (95% CI) | *P* | β | SE | Adjusted OR | (95% CI) | *P* |
| Age | 0.043 | 0.013 | 1.044 | 1.018-1.070 | 0.001 | 0.049 | 0.013 | 1.051 | 1.025-1.077 | < 0.001 |
| NIHSS score after 24 hours | 0.192 | 0.031 | 1.211 | 1.140-1.287 | < 0.001 | 0.220 | 0.031 | 1.246 | 1.173-1.324 | < 0.001 |
| mTICI ≥2b | -1.416 | 0.599 | 0.243 | 0.075-0.785 | 0.018 | -1.525 | 0.603 | 0.218 | 0.067-0.710 | 0.011 |
| sICH | | | | | / | 2.239 | 1.105 | 9.384 | 1.075-81.883 | 0.043 |
| HRR levels | -0.437 | 0.111 | 0.646 | 0.520-0.803 | < 0.001 | 0.044 | 0.084 | 1.045 | 0.886-1.232 | 0.605 |

Abbreviations: NIHSS, National Institutes of Health Stroke Scale; mTICI, modified Thrombolysis in Cerebral Infarction; sICH, symptomatic intracranial hemorrhage; HRR, hemoglobin to red cell distribution width ratio; OR, odds ratios; CI, confidence interval.

**1.2 Supplementary Table2. Multivariable analysis results for predicting death.**

| Variable | HRR after 24 hours | | | | | HRR at admission | | | | |
|---|---|---|---|---|---|---|---|---|---|---|
| | β | SE | Adjusted OR | (95% CI) | *P* | β | SE | Adjusted OR | (95% CI) | *P* |
| Age | 0.047 | 0.013 | 1.048 | 1.021-1.076 | < 0.001 | 0.039 | 0.012 | 1.040 | 1.015-1.065 | 0.001 |
| Hypertension | 0.794 | 0.339 | 2.213 | 1.138-4.304 | 0.019 | 0.835 | 0.325 | 2.305 | 1.219-4.359 | 0.010 |
| NIHSS score after 24 hours | 0.108 | 0.018 | 1.114 | 1.075-1.154 | < 0.001 | 0.127 | 0.017 | 1.136 | 1.098-1.175 | < 0.001 |
| Antiplatelet agents | -1.311 | 0.598 | 0.269 | 0.083-0.870 | 0.028 | | | | | / |
| mTICI ≥2b | | | | | / | -0.948 | 0.362 | 0.388 | 0.191-0.788 | 0.009 |
| HRR levels | -0.487 | 0.097 | 0.615 | 0.508-0.744 | < 0.001 | 0.022 | 0.062 | 1.022 | 0.905-1.154 | 0.728 |

Abbreviations: NIHSS, National Institutes of Health Stroke Scale; mTICI, modified Thrombolysis in Cerebral Infarction; HRR, hemoglobin to red cell distribution width ratio; OR, odds ratios; CI, confidence interval.

## 1.3 Supplementary Figure

**Supplementary Figure 1. Comparison of ROC curves for HRR at admission and after 24 hours according to outcomes.**

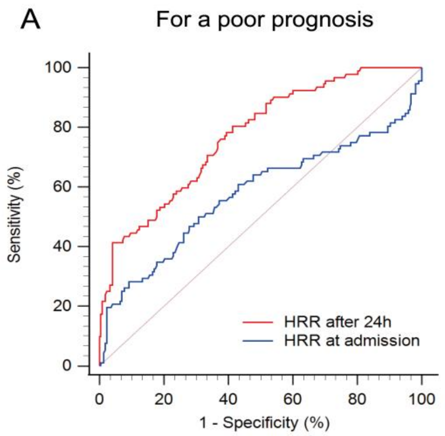


| Predictors | SE/SP (%) | ROC curves | | |
|---|---|---|---|---|
| | | AUC | 95%CI | P value |
| HRR at admission | 50.46/73.40 | 0.586 | 0.529 - 0.641 | 0.009 |
| HRR after 24h | 73.15/76.60 | 0.790 | 0.741 - 0.834 | <0.001 |

Abbreviations: HRR, Hb - to - RDW Ratio; SE, sensitivity; SP, specificity; AUC, area under the curve; ROC, receiver - operating characteristic; 95% CI, 95% confidence interval.

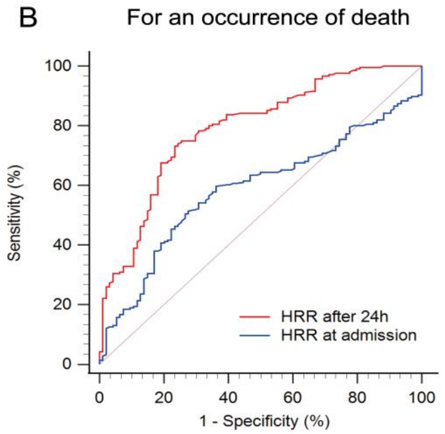


| Predictors | SE/SP (%) | ROC curves | | |
|---|---|---|---|---|
| | | AUC | 95%CI | P value |
| HRR at admission | 50.00/69.27 | 0.576 | 0.519 - 0.632 | 0.057 |
| HRR after 24h | 80.43/58.72 | 0.771 | 0.720 - 0.817 | <0.001 |

Abbreviations: HRR, Hb - to - RDW Ratio; SE, sensitivity; SP, specificity; AUC, area under the curve; ROC, receiver - operating characteristic; 95% CI, 95% confidence interval.
